# The effects of Nirvana fitness and functional training on the body appreciation of young women: non-randomized controlled trial

**DOI:** 10.3389/fpsyg.2024.1412259

**Published:** 2024-07-01

**Authors:** Rasa Jankauskiene, Vaiva Balciuniene, Renata Rutkauskaite, Simona Pajaujiene, Migle Baceviciene

**Affiliations:** ^1^Institute of Sport Science and Innovations, Lithuanian Sports University, Kaunas, Lithuania; ^2^Department of Physical and Social Education, Lithuanian Sports University, Kaunas, Lithuania; ^3^Department of Coaching Science, Lithuanian Sports University, Kaunas, Lithuania

**Keywords:** mindfulness, positive body image, exercise, women, embodiment

## Abstract

**Introduction:**

Exercise is an important intervention used to improve body image. The present non-randomized controlled trial aimed to examine the effects of Nirvana Fitness (NF) and functional training (FT) on body appreciation and its correlates in young women.

**Methods:**

Twenty-two students participated in FT, 21 in NF, and 47 in the control (CN) group. The mean age of the study participants was 22.79 ± 6.14 years. The FT and NF groups participated in sessions 2 days per week for 8 weeks, and the CN group did not participate in any sessions. All the participants were evaluated at pre- and post-intervention, filling in the Body Appreciation Scale 2 (BAS-2), Mind–Body Connection from the Physical Activity Body Experiences Questionnaire (PABEQ), Body Surveillance from the Objectified Body Consciousness Scale (OBCS), the Functionality Appreciation Scale (FAS), intrinsic exercise motivation from Behavioral Regulation in Exercise Questionnaire 2 (BREQ-2), and perceived physical fitness.

**Results:**

Significant improvements were found in terms of all outcome measures in the FT group, and improvements of body and mind connection and body surveillance in the NF group, while no improvements were observed in the CN group. FT’s effect on body appreciation appeared to operate through the improvement of the mind–body connection in the FT and NF groups and via decreased body surveillance in the FT group.

**Conclusion:**

These findings support the developmental theory of embodiment and provide initial evidence that professionally delivered FT and NF might be used as effective strategies for the promotion of positive body image in young women.

## 1 Introduction

Body image is a multidimensional construct considered to be a mental representation of appearance and body functions in perceptual, cognitive, affective, and behavioral dimensions (Cash and Smolak, 2011; Vani et al., 2021). Negative body image (body dissatisfaction) might prevent women from engaging in physical activity (PA) (More et al., 2019). Conversely, positive body image, which means accepting, appreciating, and respecting the body, is associated with various psychological and health-related lifestyle-related benefits including higher self-esteem, lower self-objectification, more intuitive eating and exercise (Tylka and Wood-Barcalow, 2015b; Sabiston et al., 2019; Linardon et al., 2022; Raspovic et al., 2023). Several previous meta-analyses have reported that exercise is associated with body and appearance satisfaction improvements of small to moderate effect size (Hausenblas and Fallon, 2006; Reel et al., 2007; Campbell and Hausenblas, 2009). Since positive body image is a much broader concept compared to body satisfaction/dissatisfaction (Tylka and Wood-Barcalow, 2015b), simply reducing body dissatisfaction or increasing body satisfaction is not enough to achieve sustainable complex changes in health-related behavior and physical and psychological well-being (Atkinson et al., 2020). Thus, developing and testing interventions that focus on positive body image and its correlates is important for the sustainable promotion of body image and healthy lifestyle in women. A recent systematic review concluded, that various strategies including exercise-related interventions targeting multiple components of positive body image can effectively promote positive body image in adults (Guest et al., 2019). However, a recent meta-analysis that targeted the intersection of body image and movement among girls and women indicated that these interventions produce small improvements in body image at post-tests, but not at follow-up (Matheson et al., 2023). Thus, it is important to continue this work.

The developmental theory of embodiment (DTE) (Piran, 2019) is one of the recommended background theories for interventions that aim to promote positive body image (Atkinson et al., 2020). The DTE describes embodiment as a construct that includes the quality of the experiences of living in the body, addressing body connection and comfort, agency and functionality, self-care, bodily desires, and freedom from body objectification (Piran, 2019). Based on DTE, engagement in pleasurable and joyful PA can strengthen the mind–body connection and the experience of positive embodiment. Embodying activities are activities that encourage awareness and attentiveness to the body, a sense of competence, and physical empowerment during PA, reducing self-objectification and body surveillance (Menzel and Levine, 2011; Menzel et al., 2019; Piran and Neumark-Sztainer, 2021). Evidence exists that PA with embodying conditions leads to a stronger mind–body connection, which is associated with lower exposure and less experience of self-objectification (constant monitoring the physical appearance of one’s body from a third-person perspective, and valuing body appearance over body functions) (Menzel et al., 2019; Piran and Neumark-Sztainer, 2021).

According to the objectification theory (Fredrickson and Roberts, 1997), women and girls’ socialization is based on the constant treatment of their bodies as sexual objects, with the focus being placed on all or part of their bodies rather than on their functional abilities. As an outcome, women and girls internalize body appearance stereotypes and start controlling their appearance from the third-person perspective. Constant body surveillance (monitoring their appearance and attractiveness from the third person perspective) is linked to negative psychological consequences such as body shame, lower focus on internal body signals, inability to experience a state of flow during PA, poorer motor performance, lower enjoyment during PA and mindfulness (Fredrickson and Roberts, 1997; Fredrickson and Harrison, 2005; Kahalon et al., 2018; Vangeel et al., 2018; Dimas et al., 2021; Gilchrist et al., 2021; Engeln et al., 2023). A longitudinal study in adolescent girls showed that body surveillance was prospectively associated with lower exercise engagement via body shame (Pila et al., 2021). However, there is some evidence that mindful PA decreases body surveillance (Cox et al., 2016a). A longitudinal study in adolescent girls showed that participation in sports was associated with lower self-objectification after one year, suggesting that sports participation might increase body functionality (Slater and Tiggemann, 2012). Body functionality describes everything that the body can do, across various domains including bodily senses and sensations, internal body processes, communication with others, working, self-care, and physical capacities (e.g., exercising) (Alleva and Tylka, 2021).

One of the main facets of positive body image is body functionality appreciation, which means not only the perception of body functionality but also includes appreciation and gratitude for it (Alleva et al., 2017; Linardon et al., 2023). Systematic analysis showed functionality appreciation was associated with greater body image, lower disordered eating, better mental health and well-being. Based on the theory, people high on body functionality should be less likely to harm themselves via unhealthy behaviors, such as dieting or unhealthy exercise, and more likely to engage in healthy and mindful eating and exercise behaviors (Alleva and Tylka, 2021). Findings of previous research showed that athletes reported higher body functionality appreciation compared to non-athletes (Soulliard et al., 2019). In their study, Swami and colleagues found that women who participated in CrossFit reported an increase in body functionality appreciation (Swami, 2019). However, the impact of other exercise types on body functionality appreciation is unknown and the present study aimed to provide more knowledge on this topic.

Exercise motivation is another important factor contributing to the association between exercise and positive body image. Based on the self-determination theory (SDT) (Ryan et al., 2009), exercising for external reasons thwarts the fulfilment of basic psychological needs such as autonomy, competence, and relatedness (Vansteenkiste et al., 2020) and prevents prolonged exercising (Ryan et al., 2009). External exercise regulations and external goals (i.e., exercising because of external or internal factors not directly related to PA) – for example, external pressures or guilt, shame, appearance, or other social recognition-related reasons - are constantly related to poorer body image (Homan and Tylka, 2014; Tylka and Homan, 2015; Hurst et al., 2017; Panão and Carraça, 2020). Conversely, research shows that intrinsic goals and exercise behaviours are related to a more positive body image (Tylka and Homan, 2015; Hurst et al., 2017; Panão and Carraça, 2020). Further, an increase in positive body image might positively affect change in internal motivation in college women (Cox et al., 2019). There is some evidence that mindfulness during PA is associated with internal exercise motivation and positive body image (Cox et al., 2016a,b; Ullrich-French et al., 2022). An intervention study using yoga showed that yoga participation might increase autonomous exercise motivation in women (Cox et al., 2020). To sum up, an increase in intrinsic motivation might be the mediator through which positive changes in positive body image occur, thus it is important to test this hypothesis.

Many studies have shown that yoga might be considered an embodying and mindful PA that positively affects body image (Cox and Tylka, 2020). Possible mechanisms through which yoga has a positive effect on body image are increased mind–body connection, self-confidence, positive embodiment, mindfulness, and decreased self-objectification (Halliwell et al., 2019; Alleva et al., 2020; Borden and Cook-Cottone, 2020; Piran and Neumark-Sztainer, 2021). Yoga is considered a mindfulness-based activity, given the mindful combination of postures or movement sequences, conscious regulation of breathing, and various techniques used to improve attentional focus on the body and mind (Cook-Cottone et al., 2020). Mindful PA is focused on the processes of becoming more internally “connected,” internally motivated, and focused on inner feelings of body and mind rather than external factors, such as one’s appearance and body acceptance from others (Calogero and Pedrotty-Stump, 2010; Menzel and Levine, 2011; Menzel et al., 2019). Based on the monitoring and acceptance theory (MAT) (Lindsay and Creswell, 2017), mindful PA is considered an exercise in which people are aware of and monitor their thoughts, feelings, and body senses and non-judgmentally accept them (Cox et al., 2016b; Ullrich-French et al., 2022). In modern wellness practice, people participate in a variety of yoga-based activities (Webb et al., 2020). One of these activities is Nirvana Fitness (NF), a system that teaches participants how to breathe correctly through a series of functional Pilates/yoga exercises. NF is a modern practice typically involving a low- to moderate-intensity combination of movement sequences, conscious breath regulation, and various techniques to improve attentional focus. It leads its participants to a slower and deeper daily breathing pattern (diaphragmatic) that should replace their shallow “default” breathing. Participants are asked to replace their shallow “default” breathing with a slower and deeper diaphragmatic pattern. Usually, yoga and yoga-based activities are practiced in stable positions and meditation without music; however, NF brings meditation into the movement flow with a stronger emphasis on rhythmic movement with music. The mindful breathing to music concept is easy to accept for modern Western populations, therefore NF has become increasingly popular worldwide.^1^ However, to date, NF as a modern type of mindfulness-based activity has not been tested for its impact on positive body image and its correlates (Guest et al., 2019), and the present study aimed to provide data on this issue.

While mindfulness-based PA has been constantly tested for its impact on positive body image, other modern group fitness exercise types lack scientific attention. An example of an activity not tested yet for its effect on body image is functional training (FT) (Guest et al., 2019). FT is a modern fitness group activity that involves dynamic movements that reflect daily life activities. The typical intensity of functional training is moderate to vigorous. FT uses a progressive programme of primarily weight-bearing multi-joint and multi-planar exercises to improve dynamic and static balance, coordination, and proprioception. This involves the integration of the nervous system, muscles that produce joint movement, as well as the muscles responsible for stabilization of the spine (core), hip, and scapulae (Beckham and Harper, 2010). Existing evidence concludes that functional training significantly impacts speed, muscular strength, power, balance, and agility in athletes (Xiao et al., 2021). FT might positively affect the body image of women since the attention in its workouts requires a strong focus on the body’s performance, but not on the appearance. Thus, it is likely that by practicing FT, body functionality might be increased, while body surveillance might be effectively decreased. These changes might positively impact positive changes in body appreciation. Therefore, it is important to test FT and its impact on positive body image.

The effect of exercise on body image might be moderated by numerous variables, including exercise mode. A meta-analysis on the associations between PA and body image showed that only the intensity of exercise was confirmed as the moderator for body image improvements, with a stronger effect from more intensive PA on body image compared to PA of lower intensity (Martin et al., 2018). However, the mode or type of exercise produced conflicting results. One meta-analysis reported a larger effect from the studies involving anaerobic exercises (i.e., weight training) compared to those involving only aerobic exercises, such as jogging (Reel et al., 2007). However, other meta-analyses found no differences in the effects of different types of exercise on the body image of men and women (Campbell and Hausenblas, 2009; Bassett-Gunter et al., 2017). The results of intervention studies comparing the effects of yoga and other modern fitness activities on women’s body image are rare and inconclusive. There is evidence that yoga and resistance exercise classes have positive effects on body satisfaction and social physique anxiety, however, it was observed that the magnitude of the change was higher after the yoga class than after the resistance class (Gammage et al., 2016). However, the opposite was found in the Turkish sample of young sedentary adults: participants of weight training sessions reported higher satisfaction with body image compared to participants of hatha yoga after 7 weeks of exercise (Taspinar et al., 2014).

Importantly, in these studies body image was measured using the negative body image concept, which describes body image as body satisfaction/dissatisfaction. However, the positive body image concept is much more complex (Tylka and Wood-Barcalow, 2015b; Swami et al., 2023). Testing the effect of various types of PAs on positive body image would provide empirical knowledge about the possibilities of using PA on sustainable body image changes that might positively impact the health-related behaviour and wellness of young women (Matheson et al., 2023). Very few studies have tested the effects of exercise programmes with different characteristics (different exercise types) on body image change (Martin et al., 2018) and only a few identified the mechanisms through which changes in body image or its correlates would occur (Cox et al., 2016a; Alleva et al., 2020). Recent systematic reviews and meta-analyses of interventions targeting the intersection of movement and body image in girls and women recommended approaching body image from a modern, holistic, and multifaceted perspective and using theoretical frameworks to inform and describe how this approach will lead to sustainable changes in girls‘and women‘s body image and movement experiences (Matheson et al., 2023).

Thus, the present study aimed to examine the effects of NF and FT on trait body appreciation and its correlates (mind–body connection, body surveillance, body functionality appreciation, perceived physical fitness, and internal exercise motivation) in a sample of young women. We also investigated the potential mechanisms that might explain the effects of NF and FT on body appreciation. In the present study, we expected that NF and FT would positively affect body appreciation and its correlates, and no changes in body image and its correlates would occur in the control group. We also hypothesized that the associations between exercise participation and positive changes in body appreciation will be mediated by positive changes in body and mind connection and body surveillance.

## 2 Materials and methods

### 2.1 Participants and ethics procedures

We conducted a non-randomized controlled trial, involving three groups, with pre- and post-test quantitative measures. This study was conducted following the Declaration of Helsinki and received ethics approval at the Lithuanian Sports University (Protocol No. SMTEK-131). All study participants provided written consent to participate in the study.

In the final stage of the recruitment, 21 students participated in NF, 22 in FT, and 47 in the CN group (Table 1). Most of the participants were studying at universities (84.5%), and the rest (15.5%) in colleges or other institutions. Of those studying in the universities, 88.2% were engaged in first-cycle studies, and the rest 11.8% in the second or third cycle. With regard to marital status, 43.3% reported being single, and 38.9% reported being married or living with a partner, 17.8% reported being in a committed relationship with a partner but living separately. Most of the participants were not employed (43.3%) or reported part-time employment (34.4%), while the rest 22.3% were in full-time employment. At baseline, the sample’s mean age was 22.79 ± 6.14 (range 18–36) years, while the mean body mass index (BMI) was 22.90 ± 4.41 (range 17.2–35.9) kg/m^2^.

**Table 1:** Descriptive characteristics of the study sample (*N* = 90).

Characteristics		*n* (%)
Group	Functional training	22 (24.4)
	Nirvana Fitness	21 (23.4)
	Control	47 (52.2)
Age (years), mean ± *SD* (range)	22.8 ± 6.1 (18–36)	
Place of studies	University	76 (84.5)
	College	12 (13.3)
	Other	2 (2.2)
University studies	First degree (bachelor’s)	67 (88.2)
	Second degree (master’s)	7 (9.2)
	Third degree (Ph.D.)	2 (2.6)
Marital status	Single	39 (43.3)
	Committed to partner, lives separately	16 (17.8)
	Lives with the partner or married	35 (38.9)
Employment status	Not employed	39 (43.3)
	Part-time	31 (34.4)
	Full-time	20 (22.3)
Body mass index	Underweight	12 (13.3)
	Normal weight	55 (61.1)
	Overweight	15 (16.7)
	Obesity	8 (8.9)
Previous participation in any sports	Yes	71 (78.9)
	No	19 (21.1)
Duration (years) of previous engagement in sports, *M* ± *SD* (range), *n* = 71	6.3 ± 4.5 (1–22)	
Previous experience in Functional Training	Yes	2 (2.2)
	No	88 (97.8)
Previous experience in Nirvana Fitness	Yes	3 (3.3)
	No	87 (96.7)
No. of exercise sessions attended, *M* ± *SD* (range)	11.1 ± 3.6 (6–17)	

### 2.2 Recruitment procedure and intervention

The recruitment of students for the intervention groups was based on voluntary participation. A flyer containing information about the intervention with an active link to the online registration form was prepared. This flyer, along with a call for registration for free exercise sessions, was disseminated through the official social media accounts of universities and colleges, and platforms of student representatives. The registration form included three groups of questions: (1) Sex, age, and contact information; (2) Previous involvement in Nirvana Fitness, Functional Training, or other sports/physical activities; (3) Health-related information such as weight, height, eating disorders, and other health conditions that could restrict exercise.

**Figure 1:**
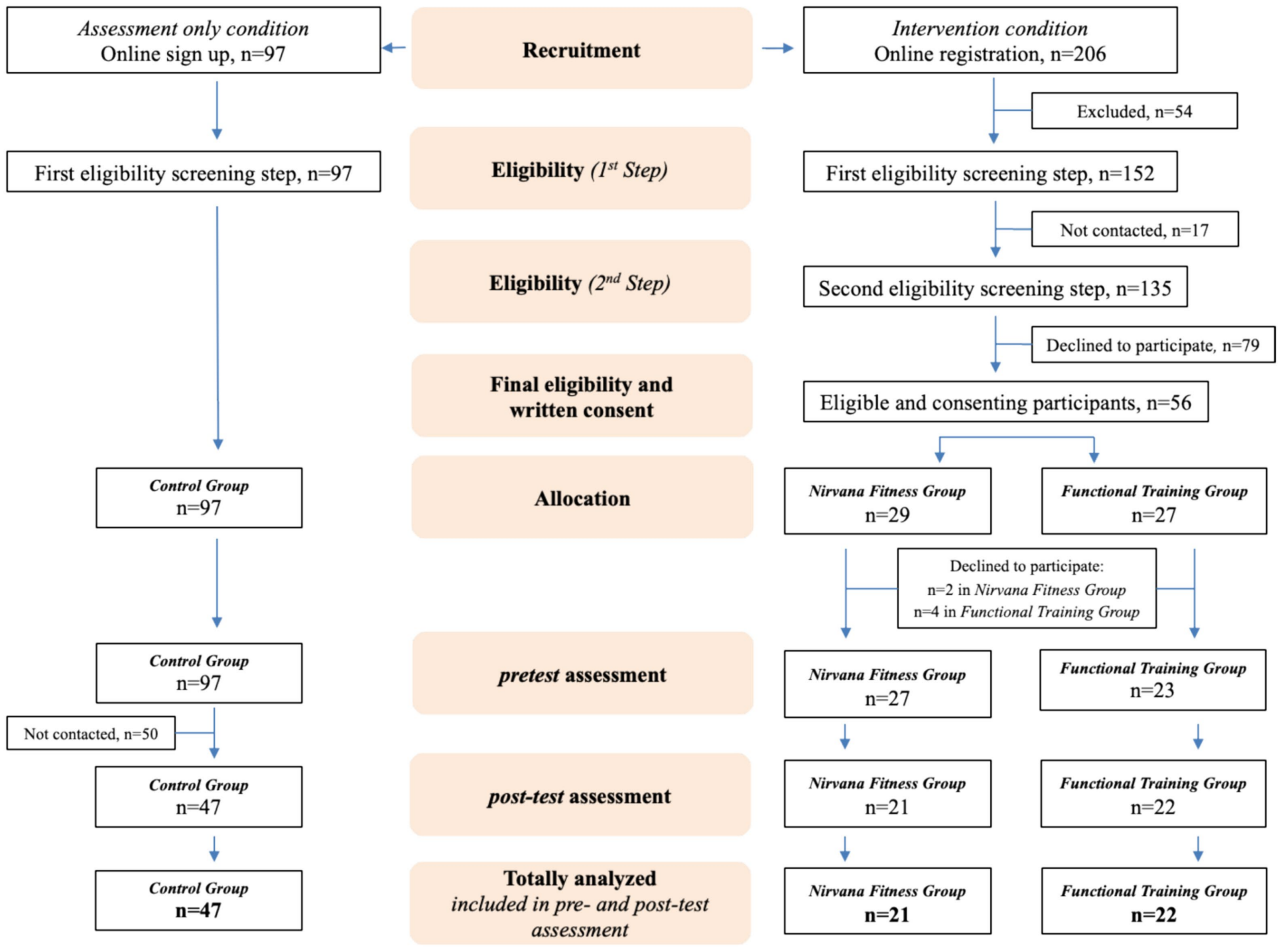
Flowchart describing participant enrolment and flow throughout the non-randomized controlled trial.

The recruitment via the online registration form lasted for 3 weeks. An initial screening for study participants’ eligibility was conducted based on the information provided in the questionnaires. Selected participants were contacted by phone for a second screening, primarily regarding their health conditions, to finalize the sample. All participants meeting the inclusion criteria received an email and a phone call reminder, inviting them to a familiarization session at the university. During this session, students received detailed information about the intervention, were allocated to either the NF or FT exercise group, and provided written informed consent to participate. Allocation to intervention groups was not random but was based on the participants’ availability regarding weekdays and times suitable for attending the exercise sessions. The flowchart presenting the recruitment of the study participants at various stages is provided in Figure 1. After the familiarization session, participants received an e-mail with information about the first exercise session and an additional phone call reminder a day before the first session.

The following eligibility criteria were enforced: being a female; up to 35 years of age; and consenting to participate in the study. The exclusion criteria were as follows: having a diagnosed eating disorder; existing musculoskeletal or other chronic conditions that can be considered barriers to exercise; participation in professional sports; and attendance of FT and/or NF in the prior 6 months.

The CN group was selected using a non-probability sampling method at the same institutions. Females were assigned to the CN group, matching them to the intervention group’s main baseline characteristics. As intervention groups underwent exercise sessions, the participants in the CN condition were aware that they were in the CN group and did not receive intervention.

Following the initial sample size calculation by an expectancy of a medium effect size (Cohen’s *d* = 0.5–0.6), with 80% statistical power and a 5% statistical significance level and using two-sided statistical tests (Dhand and Khatkatr, 2014), we aimed to recruit 25–34 female students for each of the intervention and control groups.

The 8-week intervention was conducted during the autumn semester of the 2023/2024 study year in a local health and fitness club. All three groups completed the assessments at two time points using online pre- and post-test assessments on Survey Monkey. First, a pretest assessment of the FT and NF groups was conducted immediately after the first exercise session using an e-mailed link to the questionnaire. A pretest assessment of the CN group was conducted in parallel with the FT and NF groups. Second, a post-test assessment of the FT and NF groups was conducted immediately after the last exercise session and included the same self-report instruments. A post-test assessment of the CN group was conducted in parallel with those of the FT and NF groups.

A total of 16 h were allocated for the 8-week intervention (twice a week, 1-h exercise sessions for 8 weeks), which consisted of different conditions: NF training; FT; and a CN condition. For the 8-week intervention period, the intervention structure remained stable. Each workout included a warm-up and cool-down, and the different training contents are described below.

Participants in the NF training attended two 60 min NF workouts per week. The directed 8-week NF training intervention covered four expressly defined choreographies, each of which consisted of eight sequences with four (Pilates, yoga, stretching) exercises each. Each workout included a warm-up, breathing activation, and cool-down with relaxation (Appendix A1). Participants in the FT group attended two 60 min FT workouts per week. The 8-week FT programme incorporated different types of training with determined content: stability and mobility training; body core training; body weight training and training with light resistance; resistance training, resistance circle training; interval suspension training (i.e., TRX) (Appendix A2). The CN group did not undergo any exercise sessions, and these participants followed their daily routine and regular study curriculum during the trial.

### 2.3 Measures

Body appreciation was evaluated using the Lithuanian version of the original Body Appreciation Scale 2 (BAS-2) (Tylka and Wood-Barcalow, 2015a). The instrument measures three facets of body appreciation: acceptance of one’s body; respect and care for one’s body; and protection of one’s body from unrealistic beauty standards. The BAS-2 comprises 10 items assessed on a 5-point Likert-type scale ranging from 1 (*never*) to 5 (*always*). The total BAS-2 score was the mean of the scores for all scale items. A higher score indicated greater body appreciation. The Lithuanian translation of the BAS-2, showed good psychometric properties in a sample of young people (Baceviciene and Jankauskiene, 2020). At the pretest, the scale’s internal consistency was excellent (Cronbach’s *α* = 0.96).

The mind–body connection was assessed using the five-item Mind–Body Connection subscale of the Physical Activity Body Experiences Questionnaire (PABEQ) (Menzel et al., 2019). The subscale items reflect the interaction between concepts such as thoughts, energy, physicality, awareness, and sense of self. All items were measured on a 7-point Likert-type scale ranging from 1 (*not at all true about me*) to 7 (*very true about me*). Item responses were averaged to calculate the total score on the Mind–Body Connection subscale. Higher scores reflected stronger mind–body connections and indicated that one was in a greater embodied state from physical experience. At the pretest, the subscale’s internal consistency was excellent (Cronbach’s α = 0.93). Exploratory factor analysis indicated a one-factor solution with the Eigenvalue >1, cumulatively accounting for 77.0% of the variance in the data (Kaizer–Meyer–Olkin, KMO test = 0.84, Bartlett’s Test of Sphericity 366.9, *p* < 0.001).

Body surveillance was measured using the Lithuanian version of the eight-item Body Surveillance subscale of the Objectified Body Consciousness Scale (OBCS) (McKinley and Hyde, 1996). All items were measured on a 7-point Likert-type scale ranging from 1 (*strongly disagree*) to 7 (*strongly agree*). Item responses were averaged to calculate a total body surveillance score. Higher values reflect lower body surveillance. The validity of body surveillance of the OBCS had been demonstrated in Lithuania with a sample of young women (Urvelyte, 2023). At the pretest, the internal consistency of the subscale was good (Cronbach’s α = 0.82).

Body functionality appreciation was assessed using the Lithuanian version of the original Functionality Appreciation Scale (FAS) (Alleva et al., 2017). The FAS comprises seven items assessed on a 5-point Likert-type scale, ranging from 1 (*strongly disagree*) to 5 (*strongly agree*). The total FAS score is the mean of the scores for the seven items. The higher the calculated score, the greater the appreciation of the functionality of the body. The Lithuanian-translated FAS with acceptable psychometric properties was presented by (Urvelyte, 2023). At the pretest, the scale’s internal consistency was excellent (Cronbach’s α = 0.93).

Intrinsic exercise motivation was evaluated using the four-item intrinsic motivation subscale of the Lithuanian-translated version of the original Behavioral Regulation in Exercise Questionnaire 2 (BREQ-2) (Markland and Tobin, 2004). All items were measured on a 5-point Likert-type scale, ranging from 1 (*not true*) to 5 (*very true*). The responses were averaged to calculate a total intrinsic exercise motivation score, with a higher score indicating a more intrinsic regulation of exercise. The Lithuanian translation of the BREQ-2 with adequate psychometric properties was presented in previous studies (Baceviciene and Jankauskiene, 2021). At the pretest, the subscale’s internal consistency was good (Cronbach’s α = 0.87).

Perceived physical fitness (PPF) was measured using the self-developed question, “What do you think your fitness level is? – how do you feel among your peers when it comes to climbing stairs or a hill, running, or doing physical work?” The answer options were: 1 (*too weak*); 2 (*a little weak*); 3 (*my fitness level is the same as most of my peers*); 4 (*a little stronger than others*); and 5 (*much stronger than others*). Higher values correspond to greater PFF. The same PPF assessment strategy was applied in previous studies (Baceviciene et al., 2019).

### 2.4 Statistical analyses

First, descriptive statistics were calculated per skewness, histograms, Q-Q plots, and box plots to test the distribution normality and possible continuous variable outliers. Additionally, the Shapiro–Wilk test of normality was calculated. The internal consistency of the study measures was tested by Cronbach’s α at the pretest. A Cronbach’s α over 0.65 was considered adequate (Vaske et al., 2017), while it should be noted that, generally, Cronbach’s α values are sensitive to the number of items included in the scale (Taber, 2017).

Furthermore, to compare individuals’ state experience during the first exercise session between two intervention groups, the normally distributed study variables were compared with the independent samples via a Student’s *t*-test, and the non-normally distributed with the Mann–Whitney U test. Baseline characteristics between the three groups were compared using a one-way ANOVA with the *post hoc* Bonferroni test for multiple comparisons in pairs when the homogeneity assumption was met, while non-normally distributed variables were compared with the Kruskal–Wallis H test. Eta-squared represented effect sizes for the comparisons between three groups: 0.01 and below 0.06 were considered small; 0.06 and below 0.14, medium; while ≥0.14, large (Cohen, 1992). Finally, to compare pre- and post-test results, paired-sample statistics were used (normally distributed: paired-sample Student’s *t*-test; non-normally distributed: the Wilcoxon test). The data will be presented as means and standard deviations (SDs) alongside Cohen’s *d* effect sizes, which were classified as small (0.2–0.4), medium (0.5–0.6), and large (≥ 0.8) (Cohen, 1992). The significance threshold was set at a *p*-value of < 0.05. Statistical analyses were conducted using IBM’s SPSS Statistics 29 (IBM Corp., Armonk, NY, USA).

Mediation analysis was conducted with Mplus software, Version 7.8 (Muthen & Muthen, Los Angeles, CA, USA). The intervention groups (FT vs. CN and NF vs. CN) were entered as the independent variables (X), the change in Mind–Body Connection and Body Surveillance as mediators (M), and the change in Body Appreciation as the dependent variable (Y). A bootstrapping procedure was used to test the significance of the total effect, with 10,000 bootstrap samples (Stride et al., 2016). The 95% confidence intervals for the coefficients calculated by bootstrapping methods were considered statistically significant if the confidence intervals did not include 0.

## 3 Results

Baseline sample characteristics are presented in Table 1. The mean ages of the study participants in the FT, NF, and CN groups were: 23.4 ± 2.8, 25.3 ± 9.2, and 21.4 ± 5.2, respectively (*p* < 0.001). Most of the study participants fell within the range of normal BMI (61.1%), while 25.6% were overweight/obese and 13.3% were underweight. The range of the BMI was 17.2–35.9 kg/m^2^ and did not differ significantly between groups (FT = 23.0 ± 4.6 kg/m^2^, NF = 23.9 ± 4.7 kg/m^2^, and CN = 22.5 ± 4.2 kg/m^2^, *p* = 0.372).

Most of the study participants (78.9%) reported lifetime involvement in a sport and/or PA. According to the duration of previous engagement in sports, there were no significant differences between the FT, NF, and CN groups (7.1 ± 6.3, 4.8 ± 4.0, and 6.6 ± 4.1 years, respectively, *p* = 0.351). Only two students reported previous participation in FT (one CN participant, and one FT participant), and three reported previous NF exercise sessions (two participants in the FT and one in the NF groups). The mean number of exercise sessions attended during the intervention did not differ significantly between intervention groups: FT: 11.0 ± 4.0, and NF: 11.2 ± 3.1 (*p* = 0.259).

Furthermore, Table 2 represents the comparisons of the baseline trait characteristics of the study participants, which are presented with some important differences. The NF and CN group participants reported an initially higher intrinsic exercise regulation, body functionality appreciation, body appreciation (the only difference between the FT and CN groups), and mind–body connection compared to the NF group, with medium-to-high effect sizes, while there were no differences between the CN and NF groups. Finally, the baseline PPF and objectified body surveillance scores did not differ between the three groups.

**Table 2:** The comparison of the study measures between intervention and control groups at baseline (*N* = 90).

Variables	Functional training group (*n* = 22)	Nirvana fitness group (*n* = 21)	Control group (*n* = 47)	Eta-squared	*p*
BREQ-2: Intrinsic	3.59 ± 0.92	4.27 ± 0.72*	4.13 ± 0.87*	0.09	0.020
PABEQ: Mind–Body Connection	3.40 ± 1.10	4.66 ± 1.34*	4.82 ± 1.40*	0.17	< 0.001
OBCS: Body Surveillance	3.35 ± 1.31	3.67 ± 0.80	3.97 ± 1.14	–	0.096
FAS	3.90 ± 0.46	4.45 ± 0.56*	4.35 ± 0.57*	0.14	0.002
Body Appreciation Scale 2	3.23 ± 0.82	3.67 ± 0.74	3.75 ± 0.85*	0.07	0.046
Perceived physical fitness	2.55 ± 1.10	2.90 ± 1.09	3.11 ± 0.98	–	0.102

* = as compared to the functional training group (post hoc Bonferroni test); BREQ-2, Behavioral Regulation in Exercise Questionnaire 2; PABEQ, Physical Activity Body Experiences Questionnaire; OBCS, Objectified Body Consciousness Scale; FAS, Functionality Appreciation Scale.

Finally, the changes in study characteristics during the intervention are provided in Table 3. In the FT group, all measured variables showed significant changes, including improvements in intrinsic exercise regulation, PFF, mind–body connection, functionality appreciation, and body appreciation and a decreased objectified body surveillance score, with medium-to-high effect sizes. In the NF group, only an improvement in the mind–body connection and a decline in body surveillance were observed. Conversely, no significant changes in the CN group were found.

**Table 3:** The comparison of the study variables at baseline and after the intervention (*N* = 90).

Variables	Functional training group (*n* = 22)		Cohen’s *d*	*p*	Nirvana fitness group (*n* = 21)		Cohen’s *d*	*p*	Control group (*n* = 47)		Cohen’s *d*	*p*
	Pre	Post			Pre	Post			Pre	Post		
BREQ-2: Intrinsic	3.59 ± 0.92	4.06 ± 1.05	0.58	0.009	4.27 ± 0.72	4.43 ± 0.73	–	0.228	4.13 ± 0.87	4.06 ± 0.76	–	0.470
PABEQ: Mind–Body Connection	3.40 ± 1.10	4.36 ± 1.24	1.21	< 0.001	4.66 ± 1.34	5.36 ± 1.03	0.66	0.007	4.82 ± 1.40	4.55 ± 1.32	–	0.056
OBCS: Body Surveillance	3.35 ± 1.31	4.23 ± 1.02	0.96	< 0.001	3.67 ± 0.80	4.33 ± 0.88	0.78	0.002	3.97 ± 1.14	4.27 ± 4.27	–	0.109
FAS	3.90 ± 0.46	4.31 ± 0.52	1.05	< 0.001	4.45 ± 0.56	4.52 ± 0.49	–	0.479	4.35 ± 0.57	4.34 ± 0.57	–	0.831
BAS-2	3.23 ± 0.82	3.79 ± 0.74	1.07	< 0.001	3.67 ± 0.74	3.90 ± 0.69	–	0.088	3.75 ± 0.85	3.69 ± 0.89	–	0.336
Perceived physical fitness	2.55 ± 1.10	3.09 ± 1.07	0.45	0.046	2.90 ± 1.09	3.10 ± 0.89	–	0.248	3.11 ± 0.98	3.11 ± 1.09	–	0.963

BREQ-2, Behavioral Regulation in Exercise Questionnaire 2; PABEQ, Physical Activity Body Experiences Questionnaire; OBCS, Objectified Body Consciousness Scale; FAS, Functionality Appreciation Scale; BAS-2, Body Appreciation Scale 2.

**Figure 2:**
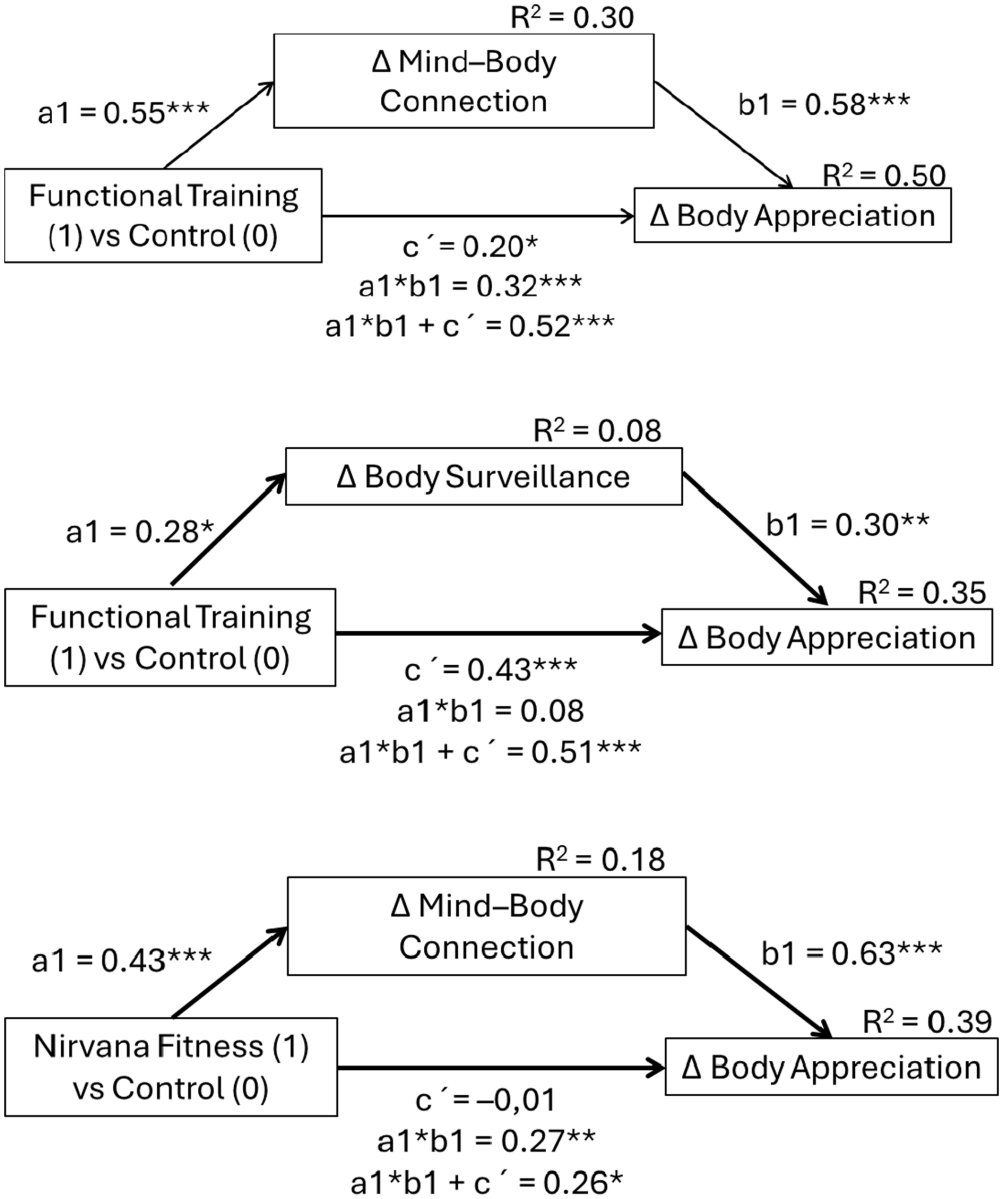
The effect of Functional training and Nirvana Fitness on body appreciation change mediated by change in the mind–body connection and body surveillance. *p < 0.05. **p < 0.01. ***p < 0.001, two-tailed. ∆ = change; coefficients are standardized; a1 = effect from the independent variable (X) on the mediator (M); b1 = effect from the M on the dependent variable (Y); c’ = direct effect from X to Y; a1 * b1 = indirect effect; a1 * b1 + c’ = total effect.

In the final step, a series of mediation models were run. Figure 2 presents the final models. The path from the FT versus CN group per the mind–body connection was significant, such that the FT group after the intervention reported higher mind–body connection scores than the CN group. Next, a higher mind–body connection predicted a stronger body appreciation. Importantly, FT had a positive direct effect on body appreciation. Next, the path of the FT group versus the CN group per objectified body surveillance was significant, such that the FT group experienced lower levels of body surveillance than the CN group. Also, positive changes in body surveillance predicted an improvement in body appreciation. Also, the FT condition had a direct positive effect on the change in body appreciation compared to the CN group. Last, a path from the NF intervention indicated an increase in the mind–body connection compared to the CN group, while the positive change in mind–body connection served as a mediator between the NF intervention and improved body appreciation. The change in body surveillance did not mediate the effect of the NF intervention on positive body image. The only significant path in the model (not shown in Figure 1) was found between a decline in body surveillance and improved body appreciation (β = 0.33, *p* = 0.001), with no NF intervention effect.

## 4 Discussion

Positive body image is a much broader concept compared to negative body image described as body (dis)satisfaction (Tylka and Wood-Barcalow, 2015b). Sustainable changes in women’s and girls’ body image, eating, and an exercise-related lifestyle require complex changes in their positive body image and its correlates, but not simply an improvement in body satisfaction (Atkinson et al., 2020). To date, only yoga as a mindful PA has mainly explored testing its impact on body appreciation, the main facet of positive body image, and its correlates (Alleva et al., 2020; Cox and Tylka, 2020). Thus, in the present study, we tested the impact of other less investigated physical activities – Nirvana Fitness and Functional Training – on positive body image (operating as trait body appreciation), body surveillance, body functionality, body and mind connection, physical fitness perception, and internal exercise motivation in a sample of student-aged women. Based on DTE, we expected that both types of exercise would produce significant changes in body appreciation and its correlates and that no changes would be observed in the control group. However, in the present study, we found that changes in body appreciation were significant only in the FT group, but not in the NF group. However, both types of PA decreased body surveillance and increased body and mind connection. Finally, participation in FT enhanced body functionality appreciation, perceived physical fitness, and internal exercise motivation. No significant changes were observed in the control group. The results of our study are in line with DTE, which states that engagement in pleasurable PA might strengthen the mind and body connection and experience of positive embodiment (Piran, 2019). Further, our findings confirm the main tenets of objectification theory (Fredrickson and Roberts, 1997), which states that body surveillance is an important factor negatively contributing to body image and its correlates in various contexts including sports. Our study provides new important empirical knowledge showing that types of exercise other than yoga might positively impact body appreciation and its correlates in young healthy women.

Only a small number of studies have shown the mechanisms through which changes in positive body image occur (Halliwell et al., 2019; Alleva et al., 2020; Matheson et al., 2023) and the present study contributes to this knowledge. In line with DTE, we found that changes in body appreciation transpired via the changes in the mind–body connection in both groups. An important new finding is that changes in body appreciation were mediated by a decrease in body surveillance in the FT group. Body and mind connection is a central construct of DTE, and the main facet of mindfulness-based physical activities such as yoga (Cook-Cottone et al., 2020). Findings of previous studies also suggested that the impact of yoga on body appreciation goes through decreased body surveillance and increased embodiment (including body and mind connection) (Alleva et al., 2020; Piran and Neumark-Sztainer, 2021). Embodying and mindful PA encourage awareness and attentiveness to the body and mind, non-judgemental acceptance of signals from the body, and different emotions and thoughts during PA (Schneider et al., 2019; Ullrich-French et al., 2022). It strengthens the body and mind connection and positively changes the direction of the focus of attention from appearance to internal body signals (Menzel and Levine, 2011).

Our results are also in line with previous studies based on the objectification theory (Kahalon et al., 2018). Specifically, positive changes in body connectedness were observed in a study testing the effect of yoga on positive body image (Halliwell et al., 2019). Reduced self-objectification mediated associations between participation in yoga and positive body image in yoga practitioners (Mahlo and Tiggemann, 2016). Decreases in self-objectification and increases in physical self-concept were observed in a prospective study of yoga participants after an 8-week period (Cox et al., 2016a). The findings of our study suggest that based on DTE and objectification theories, the mechanisms through which NF and FT positively affect body appreciation are similar to those of yoga (Alleva et al., 2020; Piran and Neumark-Sztainer, 2021).

In the present study, we also observed positive changes in body functionality appreciation – however, those changes were significant only in the FT group. Sports participation increases body functionality (Sabiston et al., 2019; Vani et al., 2021), however the impact of PA on body functionality appreciation is unclear. A previous intervention study testing the effect of yoga participation on positive body image found that yoga participation did not increase body functionality appreciation, and functionality appreciation did not mediate the associations between participation in yoga and body appreciation (Alleva et al., 2020). The findings of our study showed that NF, similarly to yoga, had no significant effect on body functionality appreciation, however FT positively impacted these changes. It is important to emphasize that FT is a different type of exercise compared to NF. It is not directly oriented to mindfulness and the intensity of typical FT is higher compared to NF. However, during FT, young healthy women have great possibilities to feel body functionality since workouts include various multi-joint movements that improve static balance, coordination, muscle strength, stabilization of the spine, hip and scapulae, and proprioception (the sense that enables you to perceive the location, movement, and action of parts of the body) (Beckham and Harper, 2010). Women, who participated in FT had no or minimal experience in FT and, contrary to the NF group, we observed a significant increase in perceived physical fitness perception at post-test. It might be that the moderate-intensity PA that was provided in the FT of the present study and challenges to the body during exercise helped women to face new body possibilities that young women were not aware of, therefore they might start valuing the body more for what it is capable of doing during exercise and in daily life. Nevertheless, we have limited opportunities to compare our findings with other studies, therefore, it is important in future studies to continue exploring the effect of FT on body functionality appreciation.

Finally, in the present study, we found that FT increased internal exercise motivation. No increases were observed in the NF group. Findings of the previous studies suggested that participation in mindful PA might increase internal exercise motivation (Cox et al., 2020). However, based on SDT, various types of exercise might positively influence internal exercise motivation if the basic psychological needs (autonomy, competence, and relatedness) of exercisers are satisfied at the interpersonal and intrapersonal levels (Vansteenkiste et al., 2020; Vasconcellos et al., 2020). In the present study, we did not assess this, thus, it is recommended for future studies. In our study, two different fitness coaches provided workouts, therefore it might be that the increase in intrinsic motivation was an outcome of the level of support for the basic psychological needs of participants but not an exercise *per se*. However, future studies should continue to explore the role of exercise in the promotion of positive body image and autonomous exercise motivation.

The present study has important limitations to be discussed. First, body appreciation, body and mind connection, functionality appreciation, and internal motivation scores were higher in the NF group compared to the FT group before the intervention. This might have impacted the results. The second important limitation is the lack of randomization. The study participants were university students with limited time for additional activities. In the present quasi-experiment, we provided only a one-time option for the workouts, therefore, the possibilities to participate in physical activity were very limited for many participants. Previous studies also reported that high attrition is common for university student samples (Harrer et al., 2020). Third, in this quasi-experiment, we asked fitness coaches to use appearance-neutral motivational language. Previous studies showed that appearance-based motivational language might impact women’s mood, body satisfaction, and body surveillance (Engeln et al., 2018). The nature of the motivational language of coaches might be a moderator, changing the associations between participation in PA and positive body image and its correlates. Thus, in future studies, it is important to address this issue. In the present study, we tested young women. In future studies, we recommend testing women of different ages. Finally, we only observed immediate changes in variables at the post-test. Measuring the stability of changes would be important to understanding the potentially long-lasting effects of NF or FT. Previous studies showed a low sustainability of changes in women and girls after exercise interventions (Matheson et al., 2023). Thus, future studies are recommended to continue this type of research.

There are also strengths of the study that should be mentioned. The attrition rates in NF and FT were low and women evaluated the exercise programmes very positively. Some participants evaluated the NF and FT experience as “something that they were not expecting from physical activity” and “life-changing.” In the present study, we used sound measures, thus the results of the study might be comparable with other studies. Finally, the present quasi-experimental study has a strong theoretical background.

The results of the present study are important for PA practice. FT and NF might be important strategies to increase positive body image in university-aged young women. Universities have good opportunities to provide PA for students. It is important to provide a spectrum of physical activities that might positively impact positive body image. The focus of attention during exercise must be directed towards body and mind and body functionality, but not body appearance; in addition, it is important to apply techniques that increase mindfulness during PA (Alleva et al., 2015). Providing various types of PA with the potential to improve body appreciation is important since it expands possibilities for young women to choose and participate in enjoyable PA. Enjoyment and satisfaction during PA is related to prolonged exercising (Ryan et al., 2009; Ntoumanis et al., 2021). Positive body image might be a background to further positive self-investments, including mindful PA and intuitive eating (Tylka and Wood-Barcalow, 2015b). Education on positive body image and PA should also be strengthened in sports science studies to increase the professionalization of sports and fitness coaches.

## 5 Conclusion

The present study adds the important new knowledge that FT positively affects body appreciation and its correlates in young women. Participation in NF and FT decreased body surveillance and increased body and mind connection. Additionally, participation in FT increased body appreciation, body functionality appreciation, perceived physical fitness, and intrinsic motivation to exercise. No significant changes were observed in the CN group. Our analysis of the possible mechanisms showed that the effect of FT and NF on body appreciation changes goes through an increased mind–body connection, while for the FT group, the effect also occurred through reduced body surveillance. These findings support the developmental theory of embodiment and provide initial evidence that professionally delivered FT and NF might be used as effective strategies for the promotion of positive body image in young women.

## Data availability statement

The data sets generated and analysed in this study can be obtained from the corresponding author.

## Ethics statement

The studies involving humans were approved by Social Research Ethics Committee of Lithuanian Sports University (Protocol No. SMTEK-131). The studies were conducted in accordance with the local legislation and institutional requirements. The participants provided their written informed consent to participate in this study.

## Author contributions

RJ: Conceptualization, Funding acquisition, Project administration, Resources, Supervision, Writing – original draft, Writing – review & editing, Investigation, Methodology, Validation, Visualization. VB: Conceptualization, Investigation, Methodology, Resources, Validation, Visualization, Writing – original draft, Writing – review & editing. RR: Investigation, Methodology, Resources, Validation, Visualization, Writing – original draft, Writing – review & editing. SP: Conceptualization, Investigation, Methodology, Resources, Writing – original draft, Writing – review & editing. MB: Conceptualization, Data curation, Formal analysis, Investigation, Methodology, Resources, Software, Validation, Visualization, Writing – original draft, Writing – review & editing, Funding acquisition.

## Footnotes

^1^ https://nirvana.fitness/instructor-locator.html
