# Supplementary material for: The effects of Nirvana fitness and functional training on the body appreciation of young women: non-randomized controlled trial
Source: Front Psychol. 2024 Jul 1;15:1412259. doi: 10.3389/fpsyg.2024.1412259 (PMC11247536; doi:10.3389/fpsyg.2024.1412259)
Supplement: Supplementary file 1 [file Table_1.docx]

**Appendix A1.**

**CONTENT OF NIRVANA FITNESS CLASSES***

| **Weeks** | **Training type** | **Aim and content** |
| --- | --- | --- |
| 1 | 1^st^ choreography | Introduction to NF system, class structure, Body oxygen check, learning the basic version of exercise sequences |
| 2 | 1^st^ choreography | Emphasis on diaphragmatic breathing and breath activation, learning modified exercise sequences |
| 3 | 2^nd^ choreography | Learning new choreography, attention to correct posture, neutral pelvic position |
| 4 | 2^nd^ choreography | Focus on relaxation, performing exercises with closed eyes, learning modified exercise sequences |
| 5 | 3^rd^ choreography | Learning new choreography, attention to a neutral spine and stable feet |
| 6 | 3^rd^ choreography | Focus on music and Theta waves, learning modified exercise sequences. Introduction to benefits of flexibility exercise. |
| 7 | 4^th^ choreography | Learning new choreography, focus on movement flow |
| 8 | 4^th^ choreography | Learning modified exercise sequences, focus on relaxation and meditation |

**Note. Each choreography consists of 8 sequences with 4 exercises each. Each class includes a warm-up and cool-down.*

**Appendix A2.**

**CONTENT OF FUNCTIONAL TRAINING CLASSES***

| **Weeks** | **Training type** | **Content** |
| --- | --- | --- |
| 1 | Stability and mobility training | Number of exercises: 6-9  Number of reps: 8-12 repetitions, a static position is held for 30 sec.  Number of sets: 1-2  Intensity: light  *Focus on correct positions (standing, kneeling, lying down) and basic exercises.* |
| 2 | Body core training | Number of exercises: 6-9  Number of reps: 8-16, a static position is held for 30 sec.  Number of sets: 1-2  Intensity: light  *Focus on core muscles and stability - mobility.* |
| 3 | Body weight training and training with light resistance | Number of exercises: 6-9  Number of reps: 8-16  Number of sets: 1-2  Intensity: light to moderate  Equipment: 2 kg dumbbells and step platform  *Exercises are performed from the easiest variation to the most difficult and are applied in regression or progression depending on how the girls feel.* |
| 4 | Resistance training, functional movement training | Number of exercises: 6-9  Number of reps 12-16  Number of sets: 2  Intensity: light to moderate  Equipment: 2-2,5 kg dumbbells, step platform, exercise ball  *Focus on coordination, balance and exercise variations are selected individually (regression or progression)* |
| 5 | Resistance circle training | Number of exercises: 6-8  Number of reps: 12-16  Number of rounds: 2-3  Intensity: moderate  Equipment: 2-2,5 kg dumbbells, 5 kg weight plate, step platform, Bosu ball, exercise ball  *Moving from exercise to exercise with a little rest in between and 1 min rest between rounds. Exercise variations are selected individually (regression or progression)* |
| 6 | Resistance training, functional movement training | Number of exercises: 6-9  Number of reps: 8-12  Number of sets: 2-3  Intensity: moderate  Equipment: 2-2,5 kg dumbbells, 5 kg Medicine ball, step platform, Bosu ball, Suspension Training system (TRX)  *Exercise variations are selected individually (regression or progression). In the first training of the week are more emphasis on the upper part of the body, in the second training - on the lower part of the body.* |
| 7 | Interval Suspension Training - TRX | Number of exercises: 8  Number of reps: 8-16  Number of sets: 3  Intervals: 45 sec exercise, 30 sec rest  Intensity: moderate  Equipment: Suspension Training system (TRX)  *Exercise variations are selected individually (regression or progression).* |
| 8 | Functional training | Number of exercises: 8-10  Number of reps: 8-16  Number of sets: 3  Intensity: moderate  Equipment: resistance band, exercise ball, kettlebell  *Focus on body functionality, awareness, breathing, coordination, and balance.* |

**Note. Each class includes a warm-up and cool-down.*
